# Lesions of the Teeth in Locomotor Ataxy

**Published:** 1883-01

**Authors:** 


### Lesions of the Teeth in Locomotor Ataxy.

The *Medical Times and Gazette* says: At the meeting of the French Association for the Advancement of Science, on August 30th, a communication was made by M. Th. David, upon lesions of the teeth in locomotor ataxy. The paper was based upon the observation of a single case, and the following are the most important of the conclusions arrived at from an attentive study of it: The alteration consists of a rapid decay of the anterior part of the crown of almost all the teeth. The altered substance assumes the consistence of touchwood and a reddish color. The enamel still retained its polish, but not its hardness. Beneath those parts the pulp had produced a new layer of secondary dentine, and in most of the front teeth the pulp cavity was filled up. These alterations had nothing in common with caries, and must be referred to nutritive disturbance resulting from the lesion of the central nervous system. The changes are analogous to those which have already been observed to take place in the nails in the course of locomotor ataxy; they would thus establish a pathological relationship between organs already connected by a common epithelial origin. Locally, these alterations recognize for their immediate cause a functional disturbance or a lesion of the dental pulp. The atrophy which has been shown to exist would be quite comparable to that which is observed in the eye under similar circumstances. Whence the final conclusion that we must attribute to the dental pulp the physiological significance of a sensory organ.

